# A New Dataset of Spermatogenic *vs.* Oogenic Transcriptomes in the Nematode *Caenorhabditis elegans*

**DOI:** 10.1534/g3.114.012351

**Published:** 2014-07-24

**Authors:** Marco A. Ortiz, Daniel Noble, Elena P. Sorokin, Judith Kimble

**Affiliations:** * Department of Biochemistry, University of Wisconsin-Madison, Madison, Wisconsin 53706; † Graduate Program in Cellular and Molecular Biology, University of Wisconsin-Madison, Madison, Wisconsin 53706; ‡ Howard Hughes Medical Institute, University of Wisconsin-Madison, Madison, Wisconsin 53706

**Keywords:** germline sex, RNA-Seq, *C. elegans*, splicing, Genetics of Sex

## Abstract

The nematode *Caenorhabditis elegans* is an important model for studies of germ cell biology, including the meiotic cell cycle, gamete specification as sperm or oocyte, and gamete development. Fundamental to those studies is a genome-level knowledge of the germline transcriptome. Here, we use RNA-Seq to identify genes expressed in isolated XX gonads, which are approximately 95% germline and 5% somatic gonadal tissue. We generate data from mutants making either sperm [*fem-3(q96)*] or oocytes [*fog-2(q71)*], both grown at 22°. Our dataset identifies a total of 10,754 mRNAs in the polyadenylated transcriptome of XX gonads, with 2748 enriched in spermatogenic gonads, 1732 enriched in oogenic gonads, and the remaining 6274 not enriched in either. These spermatogenic, oogenic, and gender-neutral gene datasets compare well with those of previous studies, but double the number of genes identified. A comparison of the additional genes found in our study with *in situ* hybridization patterns in the Kohara database suggests that most are expressed in the germline. We also query our RNA-Seq data for differential exon usage and find 351 mRNAs with sex-enriched isoforms. We suggest that this new dataset will prove useful for studies focusing on *C. elegans* germ cell biology.

Germ cell biology is central to reproduction and fertility. The nematode *Caenorhabditis elegans* is a well-established model for genetic and molecular analyses of germline sex determination (Ellis and Schedl 2007; Kimble and Crittenden 2007), progression through the meiotic cell cycle (Rog and Dernburg 2013), and gametogenesis (Greenstein 2005; L’Hernault 2006). Moreover, *C. elegans* was the first metazoan with a fully sequenced genome (*C. elegans* Sequencing Consortium 1998) and a central player in ModENCODE (Gerstein *et al.* 2010). Therefore, *C. elegans* is poised to serve as a model to analyze germ cell biology at a comprehensive systems level.

State-of-the-art transcriptome data lie at the foundation of virtually any modern study of biological regulation. To this end, Reinke *et al.* (2004) reported a pioneering analysis of spermatogenic and oogenic transcriptomes. This now classic study relied on mRNAs extracted from whole animals, custom-spotted microarrays, and a 2003 version of the *C. elegans* genome annotation. Other studies have also generated the following relevant transcriptomes: maternal RNAs, which can be used as a proxy for a subset of oogenic RNAs (Baugh *et al.* 2003); germline-specific RNAs obtained from gonads extracted from adult wild-type hermaphrodites and subjected to serial analysis of gene expression (SAGE) (Wang *et al.* 2009); and RNAs in isolated mature sperm (Ma *et al.* 2014). In addition, a study identified several hundred mRNAs whose expression depends on the sperm-specific transcription factor SPE-44 (Kulkarni *et al.* 2012); a proteomics study discovered proteins in isolated mature oocytes (Chik *et al.* 2011); and RNA immunoprecipitation studies identified RNAs associated with RNA-binding proteins in adult oogenic germlines [FBF-1 (Kershner and Kimble 2010); GLD-2 and RNP-8 (Kim *et al.* 2010); GLD-1 (Jungkamp *et al.* 2011); EFL-1 and DPL-1 (Kudron *et al.* 2013)]. However, the classic analysis of Reinke *et al.* (2004) remains the only dataset available focusing on spermatogenic *vs.* oogenic transcriptomes.

As background for the current work, *C. elegans* develops as either XX hermaphrodites or XO males; XX hermaphrodites make sperm as larvae and oocytes as adults, whereas XO males make sperm only and continuously. Because nematode XX hermaphroditism evolved recently, closely related species remain gonochoristic (XX females and XO males), and elimination of a single gene, *fog-2* (feminization of germline), transforms *C. elegans* into a gonochoristic strain with XX females and XO males (Schedl and Kimble 1988). A single gonadal arm in adult *C. elegans* XX hermaphrodites possesses ∼1000 germ cells, with stem cells at one end and differentiating gametes at the other; each isolated gonad also possesses ∼25 somatic gonadal cells. Many existent sex determination mutants affect the germline, including nonconditional and temperature-sensitive alleles of varying strengths and tissue specificities. Of particular relevance to this work are temperature-sensitive *fem-3(gf)* and homozygous *fog-2* mutants. XX *fem-3(gf)* mutants make no oocytes, but instead produce sperm continuously in a hermaphrodite/female soma (Barton *et al.* 1987); by contrast, *fog-2* mutants make no sperm but make oocytes continuously in an equivalent soma (Schedl and Kimble 1988).

Our analysis of spermatogenic and oogenic transcriptomes begins with XX animals possessing germlines of the opposite sex but housed in somas of equivalent sex, RNAs extracted from isolated gonads, RNA-Seq data based on eight biological replicates, and the most recent version of the *C. elegans* reference genome annotations available in Ensembl (Flicek *et al.* 2014). Other details of our experimental design differ from those used previously, as outlined in *Results*. Where possible, we used the Kohara *in situ* hybridization database (NEXTDB: *nematode.lab.nig.ac.jp/*) to validate RNAs that previously had not been identified as expressed in the germline or previously had not been annotated as spermatogenic or oogenic. We compared our data to relevant datasets mentioned above and analyzed our data for alternative splicing. We suggest that this new dataset will prove useful in combination with other datasets for studies of germline regulation and gamete differentiation.

## Materials and Methods

### *C. elegans* strains

We used two homozygous mutant strains: *fem-3(q96gf) IV*, which is temperature-sensitive, and *fog-2(q71) V*. The *fem-3(q96gf)* stocks were maintained at 15° and experimental animals grown at 22°; *fog-2* mutants were maintained at 22°. For an immunostaining control, we used wild-type Bristol strain N2, also grown at 22°.

### Dissection of gonads for immunostaining

Synchronized young adults (0–2 hr past the L4 to adult molt) were cut just behind the pharynx in PBS-Tween (0.1% Tween20) with 0.25 mM Levamisole; dissected animals were fixed in 3% paraformaldehyde, 0.1 M K_2_HPO_4_ for 30 min and permeabilized in 100% methanol at −20° for 30 min. Samples were washed three times in PBST and blocked in PBST plus 0.5% BSA for 30 min at room temperature. Samples were incubated with primary antibodies at 4° overnight in PBST plus 0.5% BSA at the following dilution: mouse anti-SP56 (1:100) (gift from S. Strome) and rabbit anti-RME-2 (1:500) (gift from B. Grant). They were then incubated with Cye3 and FITC conjugated secondary antibodies (Jackson ImmunoResearch), both at a 1:1000 dilution in PBST plus 0.5% BSA, for 1 hr at room temperature. Finally, samples were mounted on slides in VectaShield containing DAPI to visualize DNA and imaged with a Zeiss Axioimager microscope.

### Isolation of gonads for RNA extraction

Synchronized young adults (0-2 hr past the L4 to adult molt) were first cut behind the pharynx as described above. Gonadal arms were then cut at or near the spermathecae to isolate them from the carcass. Total RNA was extracted from the gonads using TRIzol (Invitrogen) and RNeasy Mini Kit (Qiagen) following the manufacturer’s instructions.

### Replicates and sequencing

We generated eight independent samples for *fog-2* and another eight for *fem-3*. Each sample contained approximately 30 gonadal arms and most (14/16) had a total RNA concentration of 20–34 ng/ml. The University of Wisconsin Biotechnology Center prepared libraries for each sample using the TruSeq Illumina sequencing protocol, which includes mRNA purification (poly-A selection) and fragmentation, cDNA synthesis, end repair, adapters ligation, and DNA fragment enrichment. Each library was bar-coded and sequenced in four different lanes to obtain single-end 101-bp reads using Illumina HiSeq2000. We obtained more than 36 million reads of high-quality score (>35 mean quality score) on average per sample. All sequencing data are available in the National Institutes of Health Gene Expression Omnibus database under accession number GSE57109.

### Transcript analysis

We used TopHat2 v2.0.11 (with −g 1 option) (Trapnell *et al.* 2012) to align reads to the *C. elegans* reference genome (WBcel235.75.fa) and gene annotations (WBcel235.75.gtf) in WormBase WS240 (Ensembl) (Flicek *et al.* 2014). For compatibility to feature-counting software, we created sorted and indexed SAM versions of the BAM files (SAMtools) (Li *et al.* 2009). To create a read-count dataset, we processed SAM files with python scripts described elsewhere (Anders and Huber 2010). Our cutoff was two mapped reads per gene for each of eight replicates or a minimum of 16 total reads per gene, applied to each mutant independently. Genes with ambiguous annotations or fewer reads (<16 reads/gene) were removed. To identify differentially expressed transcripts, we used R/Bioconductor package DESeq, a common method to evaluate differential expression (Anders and Huber 2010; Rapaport *et al.* 2013). DESeq performs normalization by applying a scaling factor to each sample; this scaling factor is the median calculated from the ratios of read counts for each gene to the geometric mean of all samples (Anders and Huber 2010; Rapaport *et al.* 2013). We also determined abundance as rpkm using Cufflinks v2.1.1 (with −N, −u, and −b options) (Trapnell *et al.* 2012) (Supporting Information, Table S1). In total, we found 10,754 unambiguously expressed genes with at least 16 uniquely high-quality aligned reads. For data plotting, we used ggplot2 R/Bioconductor package (Wickham 2009).

### Exon usage analysis

We used TopHat v2.0.11 (−m 2 −g 1 and −G options) to align reads to the *C. elegans* reference genome (WS235). We used Cufflinks v2.1.1 (with −N, −u, and −b options) (Trapnell *et al.* 2010; Trapnell *et al.* 2012) to assemble isoforms and measure isoform expression. For analysis of differential exon usage between spermatogenic and oogenic gonads, we used the DEXSeq package for R/Bioconductor (Anders *et al.* 2012). We obtained reads per exon from the alignment files using a script accompanying the DEXSeq package. For an exon to be scored significantly different, we set a false discovery rate of <1% and a minimum fold change requirement of two-fold. We also set a minimum expression threshold of two reads per exon. We used gene annotations in WS240 and a custom script to compare our differential exon results with annotated alternative exon usage. Custom scripts were also used to count the number of exon junction–spanning reads for the alignment files to determine whether alternative exon usage was in a coding or noncoding region and to discriminate types of alternative splicing observed. The UCSC Genome Browser ce10 (www.ucsc.genome.edu) was used to visualize alternative splicing of WIG files that were converted from BAM files using SAMtools mpileup (SAMtools) (Li *et al.* 2009) and a custom script.

## Results and Discussion

### Experimental design

**Figure 1:**
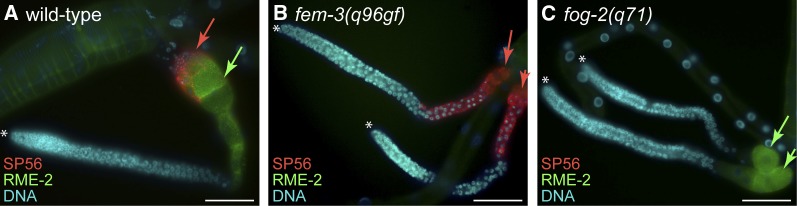
Immunocytochemistry of sex-specific gamete markers in wild-type and mutants used in this work for RNA-Seq. (A–C) Extruded gonads from young adult XX worms (L4/A molt+2 hr), all raised at 22°; each image merges staining of the α-SP56 sperm-specific marker (red), α-RME-2 oocyte-specific marker (green), and DAPI DNA marker (blue). Asterisk marks distal end of gonad; red arrows mark SP56; green arrows mark RME-2. (A) Wild-type hermaphrodite gonad with both sperm-specific and oocyte-specific markers. (B) Sperm-only *fem-3(q96gf)* gonads stain only with sperm-specific marker (red). (C) Oocyte-only *fog-2(q71)* gonads stain only with oocyte-specific marker (green). Scale bars: 50 μm.

Many mutants affecting gamete sex must be grown at 25°, thus reducing fertility even in wild-type animals (Hirsh *et al.* 1976). We avoided growth at 25° by using *fog-2(q71)* mutants, whose XX germline makes oocytes and only oocytes at all standard growth temperatures (Schedl and Kimble 1988), and the strong *fem-3(q96gf)* allele (Barton *et al.* 1987), which makes sperm and only sperm when raised at 22° even 3 days into adulthood (100%; n = 150). To ensure germline sex transformation of these two mutants, we stained for sex-specific germline markers, SP56 for spermatogenic germlines (Ward *et al.* 1986) and RME-2 for oogenic germlines (Grant and Hirsh 1999). For wild-type animals grown at 22°, staining was as expected with SP56-positive sperm and RME-2-positive oocytes (Figure 1A). More importantly, *fem-3(q96)* sperm-only adults raised at 22° possessed SP56 but not RME-2 (Figure 1B), and *fog-2(q71)* oocyte-only adults raised at 22° had RME-2 but not SP56 (Figure 1C). Yet these two XX mutants have morphologically indistinguishable somatic tissues, including the somatic gonad (Barton *et al.* 1987; Schedl and Kimble 1988). We note that use of mutants was essential for this analysis and caution that a mutant transcriptome may include changes not found in wild-type and that XX sperm are only a proxy for XO sperm.

Two other features were specific to our analysis of spermatogenic *vs.* oogenic transcriptomes. First, we isolated gonads and discarded the main body and intestine; an isolated gonad contains approximately 1000 germ cells but many fewer somatic gonadal cells (1 DTC, 10 sheath, and some portion of the 26-celled spermatheca). Gonad isolation therefore removes most somatic RNAs (*e.g.*, hypodermis, nerve, muscle, intestine) but still includes approximately 25 somatic gonadal cells. Second, we isolated gonads from young adults only 0–2 hr after the molt from L4 to adulthood. This stage harbors fully differentiated gametes but has not begun to accumulate unused gametes, which affect germline physiology (*e.g.*, mitotic index) (Jaramillo-Lambert *et al.* 2007; Morgan *et al.* 2010).

### Genes expressed in gonadal transcriptome

**Figure 2:**
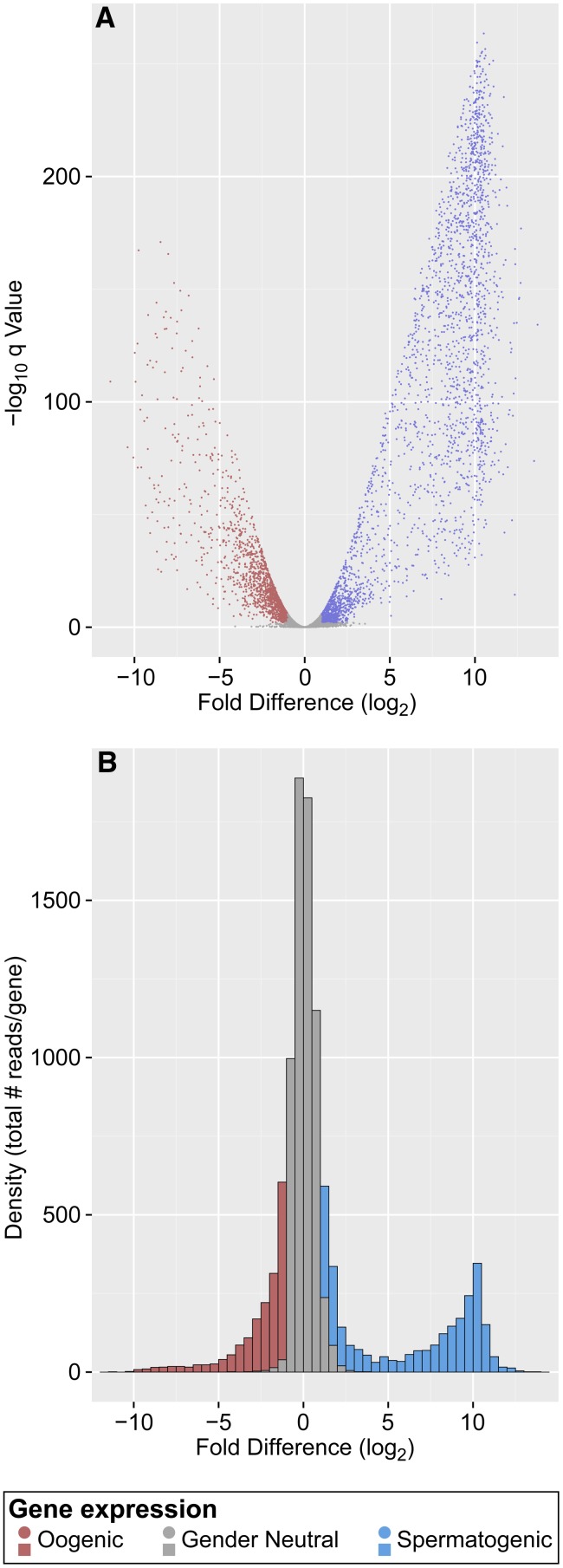
Identification of genes expressed in spermatogenic and oogenic gonads. (A) Volcano plot of all genes expressed in *fem-3(gf)* spermatogenic and *fog-2* oogenic gonads, showing fold changes in abundance on the X-axis and significance on the Y-axis. Colors indicate enriched expression in spermatogenic gonads (blue), oogenic gonads (red), or neither (gender-neutral) (gray). (B) Histogram of all genes expressed in *fem-3(gf)* spermatogenic and *fog-2* oogenic gonads, showing fold changes in expression abundance with color coding as in (A).

We prepared and sequenced polyadenylated RNAs from eight biological replicates of each mutant [*fem-3(gf)* and *fog-2*], with 30 isolated gonads per replicate. Using TopHat (Trapnell *et al.* 2012), >90% of sequence reads could be mapped uniquely to the most recent version of the *C. elegans* genome (see *Materials and Methods*). A gene was scored as expressed when 16 or more reads mapped to that gene. Expression abundance was measured in normalized total read counts per gene. Because transcript isoforms were ignored for this first analysis, we discuss the data in terms of “genes expressed” rather than “transcripts expressed.” For quality reference, 97% of the reads mapped to the annotated transcriptome; of those, 1.7% mapped partly to noncoding sequences and partly to coding sequences and 0.3% fell in ambiguous gene sequence annotations. In this way, we identified totals of 10,733 genes expressed in *fem-3(gf)* gonads, 10,631 in *fog-2* gonads, and 10,610 shared (Figure 2A). Together, a total of 10,754 mRNAs comprise the polyadenylated transcriptome of these XX gonads.

We compared our dataset with a previously published dataset of 4699 mRNAs detected in isolated wild-type adult hermaphrodite gonads using SAGE (Table 1). Most SAGE-identified genes were also found in our work (92%; 4323/4699). We also compared our dataset with lists of germline transcripts deduced by comparing “soma plus germline” to “soma-only” transcriptomes. Reinke *et al.* (2004) found 3145 germline RNAs in this manner from microarray data, whereas Wang *et al.* (2009) found 1063 using SAGE. Again, most germline RNAs previously reported were also identified in this work (84%; 2637/3145 and 95%; 1017/1063). The design differences among these various studies (Table 1 and Table 2) were so extensive that the main conclusion is that our data include most genes previously reported as expressed in the gonad and doubles their number.

**Table 1:** Total genes expressed in isolated gonads

Paper	Strain	Stage	Gamete	Temperature	Method	Genome Version	Total # Genes Expressed	% Overlap*^a^*
Wang *et al.* (2009)	XX wild-type hermaphrodite	Adult (L4 + 18 hr)	Sperm and oocytes	20°	SAGE	WS160 (2006)	4699	92%
This work	XX *fem-3(q96)* and *XX fog-2(q71)*	Adult (L4 molt + 2 hr)	Sperm and oocytes	22°	RNA-Seq	WS240 (2014)	10,754	NA

NA, not applicable.

a Percent of total transcripts identified by Wang *et al.* (2009) that were also found in this work.

**Table 2:** Experimental design of studies of sex-enriched expression

Features	Reinke *et al.* (2004)	This Work
Sperm-only mutant	*fem-3(q23gf)*	*fem-3(q96gf)*
Oocyte-only	*fem-1(hc17)*	*fog-2(q71*)
Stage	Young adult	L4/A molt + 2 hr
Growth temperature	25°	22°
RNA source	Whole animal	Isolated gonad
Methods for RNA detection	Microarray	RNA-Seq
Genome version	WS90	WS240
Statistical threshold	p-value = 0.01	Adjusted p-value = 0.01

#### Gene expression enriched in spermatogenic vs. oogenic gonads:

We next identified genes whose expression was enriched in spermatogenic *vs.* oogenic gonads. To this end, we used DESeq to compare the 10,733 genes expressed in *fem-3(gf)* spermatogenic gonads with the 10,631 in *fog-2* oogenic gonads. Using thresholds of a two-fold difference in abundance and a false discovery rate of 1%, we identified 2748 genes with expression enriched in spermatogenic gonads and 1732 enriched in oogenic gonads; the remaining 6274 were not enriched in either spermatogenic or oogenic gonads (Figure 2, Table 3, and Table S1). We refer to these as spermatogenic, oogenic, and gender-neutral genes, respectively. All spermatogenic, oogenic, and gender-neutral genes are labeled as such and listed in an Excel searchable format in Table S1, column H. Table 4 shows 12 representative genes previously determined experimentally to have gender-neutral or sex-biased germline expression.

**Table 3:** Sex-enriched expression

	Reinke *et al.* (2004)	This Work 2014	Overlap*^a^*
Spermatogenesis-enriched genes	865	2748	98%
Oogenesis-enriched genes	1030	1732	37%
Gender-neutral	1250	6274	83%

a Percent transcripts in Reinke *et al.* (2004) also found in this work.

**Table 4:** Representative genes with known sex bias expression

Gene Name	Sex-Biased Expression	Original Reference for Sex Bias	*fog-2* Expression Value *^a^*	*fem-3* Expression Value*^a^*	log_2_ *fog-2/fem-3* Expression Value*^a^*		Adjusted p-value (FDR)*^a^*	
*cpb-1*	Spermatogenic	Luitjens *et al.* (2000)	16.0E+02	55.0E+02	1.77	5.68E−12		
*fog-1*	Spermatogenic	Luitjens *et al.* (2000)	6.26E+02	38.8E+02	2.63	1.08E−27		
*fog-3*	Spermatogenic	Chen *et al.* (2000)	0.195E+02	56.9E+02	8.18	1.7E−189		
*spe-44*	Spermatogenic	Kulkarni *et al.* (2012)	5.32E+02	47.8E+02	3.16	1.13E−48		
*oma-1*	Oogenic	Detwiler *et al.* (2001)	90.9E+02	40.5E+02	−1.16	8.84E−08		
*pie-1*	Oogenic	Tenenhaus *et al.* (1998)	34.6E+02	4.84E+02	−2.83	5.78E−41		
*rme-2*	Oogenic	Grant and Hirsh (1999)	184.E+02	33.5E+02	−2.46	1.22E−38		
*tra-2*	Oogenic	Okkema and Kimble (1991)	21.5E+02	0.574E+02	−1.90	5.78E−20		
*him-3*	Gender-neutral	Zetka *et al.* (1999)	15.8E+02	28.9E+02	0.88	5.76E−06		
*ima-1*	Gender-neutral	Geles and Adam (2001)	44.7E+02	26.2E+02	−0.76	2.01E−04		
*ima-3*	Gender-neutral	Geles and Adam (2001)	80.4E+02	75.8E+02	−0.08	5.26E−24		
*spo-11*	Gender-neutral	Dernburg *et al.* (1998)	18.7E+02	15.4E+02	−0.63	4.93E−03		

a This work.

A comparison of our datasets (spermatogenic, oogenic, and gender-neutral genes) with those of a similar but earlier study (Reinke *et al.* 2004) reveals considerable overlap between spermatogenic and gender-neutral genes, but less with oogenic genes (Table 3). Only 37% of oogenic genes were found in our oogenic gene list. Where were the missing 63%? The majority were in the gender-neutral gene list (99%). Why were so many “oogenic” genes in the study by Reinke *et al.* (2004) “gender-neutral” genes in our study? One possible explanation is that our work queried very young adult gonads with only a few mature oocytes, whereas Reinke *et al.* (2004) used older adults with many mature oocytes. Another explanation is that our study had greater sensitivity. Both explanations likely have some validity. We blindly surveyed expression of all the Reinke *et al.* (2004) study’s oogenic genes in the NEXTDB database and found 582 genes with unambiguous *in situ* hybridization data; of these, 42% (242/582) were enriched for germline expression in adult hermaphrodites compared with L4s, consistent with their oogenic classification (Table S1, column I), whereas 58% (243/582) were expressed at approximately the same level in both larval and adult germlines. The percentage of the Reinke *et al.* (2004) study’s oogenic genes validated as enriched during oogenesis according to NEXTDB (42%) is therefore similar to the percentage overlap between oogenic genes found here and that work (37%) (Table 3). We suggest that the datasets are comparable but not identical.

**Figure 3:**
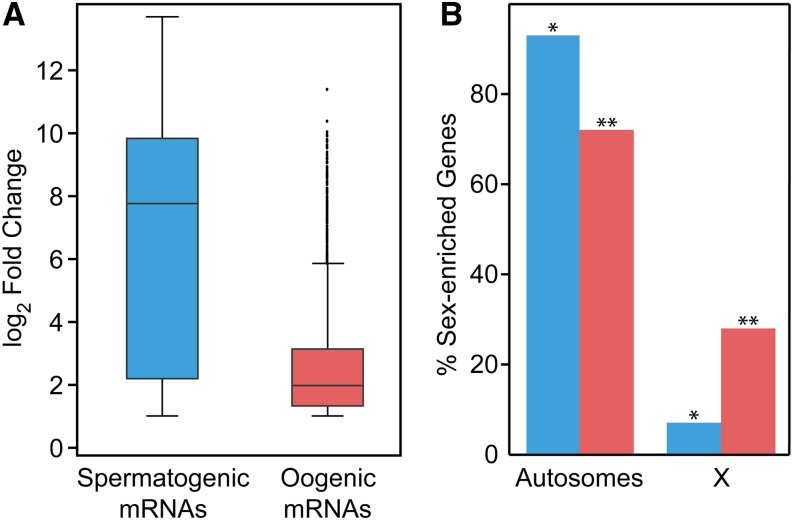
Analyses of spermatogenic and oogenic-enriched mRNAs/genes. (A) Box plot of expression fold changes for spermatogenic-enriched (blue) and oogenic-enriched (red) mRNAs, with whiskers indicating outliers. (B) Bar graph showing percentage of genes encoding spermatogenic-enriched (blue) or oogenic-enriched (red) mRNA on autosomal (left) and X (right) chromosomes. Significance of enrichment or depletion was calculated using Hypergeometric distribution with comparisons between the number of genes with sex-enriched expression on autosomal or X chromosomes and the total number of gonad-expressed genes on autosomal (9476 genes) or X chromosomes (1274 genes). *p-value < 10^−24^; **p-value < 10^−93^.

We also compared our data with recent studies of sex-biased gene expression and evolution (Thomas *et al.* 2012; Albritton *et al.* 2014). In agreement with those works, we found that the average fold change in expression of spermatogenic genes was greater than that of oogenic genes (Figure 3A). We also found that oogenic genes were significantly enriched compared with total gonadal genes on chromosome X (28% oogenic *vs.* 22% gonadal on X) but underenriched on autosomes (72% *vs.* 78% on autosomes); conversely, spermatogenic genes were significantly underenriched on chromosome X (7% spermatogenic *vs.* 12% gonadal on X) but enriched on autosomes (93% *vs.* 88% on autosomes) (Figure 3B, see legend for p-values), confirming previous results (Reinke *et al.* 2000, 2004). Finally, those previous studies found more spermatogenic genes than oogenic genes, in agreement with our results (Figure 2, Figure 3A, and Table S1). Therefore, recent studies using RNA-Seq from whole worms are consistent with this analysis of gonadal sex-biased gene expression.

We hypothesized that genes involved in common germline processes, such as meiosis, would be enriched in the gender-neutral expression category. To test this idea, we searched for genes involved in meiosis according to Gene Ontology classification and RNAi experiments reported in WormBase. Our gender-neutral list included 82% (231/281) of those genes with meiosis-related functions (Table S2). Thus, meiotic cell cycle genes are enriched in our gender-neutral dataset compared with sex-enriched datasets.

#### Gonadal genes identified specifically in this study:

Using the NEXTDB database, we explored the tissue expression of gonad-expressed mRNAs identified in this work but not in previous studies of isolated gonads (Wang *et al.* 2009) or germline expression (Reinke *et al.* 2004). NEXTDB archives *in situ* hybridization results for approximately half the annotated *C. elegans* genes and includes unambiguous staining patterns for 1522 of the 2567 genes found specifically in this study. Of these 1522 staining patterns, 92% of spermatogenic (178), 90% of oogenic (294), and 74% of gender-neutral (743) genes were expressed in the germline tissue according to NEXTDB (Table S1, column J). Moreover, a sampling of genes was scored for gender expression. Most genes classified as sperm-enriched were visibly more abundant in L4 spermatogenic than adult oogenic germlines (69%; n = 103); most classified as oocyte-enriched were visibly more abundant in adult than L4 germlines (75%, n = 216); and most classified as gender-neutral were found at both stages with no obvious visible difference in abundance (78%; n = 363). Therefore, our dataset provides a new source for genes expressed in the germline.

#### Other comparisons:

**Figure 4:**
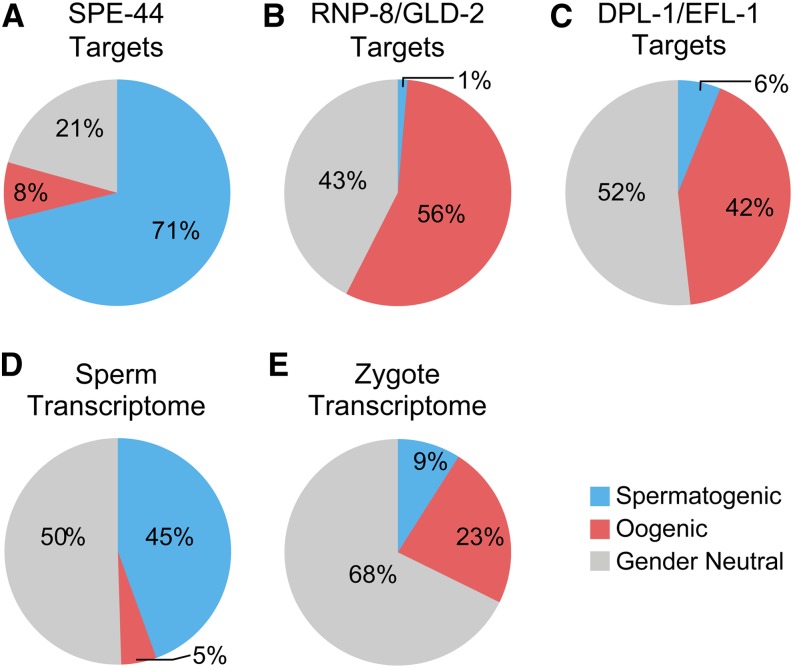
Dataset comparisons. (A–E) Pie charts with overlaps between other relevant datasets, as noted in figure, and this work’s spermatogenic (blue), oogenic (red), and gender-neutral (gray) datasets. See text for explanation and references.

We also compared our spermatogenic, oogenic, and gender-neutral datasets with several others (Figure 4). First, we compared them to lists of target genes or target mRNAs of crucial gamete regulators. The SPE-44 transcription factor drives spermatogenesis with many predicted target genes (Kulkarni *et al.* 2012). Most *spe-44* predicted targets were found in our list of spermatogenic genes (475/668) but not in our list of oogenic genes (55/668) (Figure 4A). GLD-2/RNP-8 and EFL-1/DPL-1 heterodimers drive the process of oogenesis (Kim *et al.* 2009; Kudron *et al.* 2013). Many GLD-2/RNP-8 (178/317) and EFL-1/DPL-1 (130/309) predicted targets were on our list of oogenic genes, and the majority in a combined list of oogenic plus gender-neutral genes [GLD-2/RNP-8 (313/317); EFL-1/DPL-1 (290/309)], whereas overlap with spermatogenic genes was low [GLD-2/RNP-8 (4/317)**;** EFL-1/DPL-1 (19/309)] (Figure 4, B and C).

Second, we compared our datasets with transcriptomes obtained from isolated mature sperm (Ma *et al.* 2014) or zygotes (Baugh *et al.* 2003). Both contained many of our gender-neutral expression gene set. However, the mature sperm transcriptome contained almost half of our spermatogenic gene dataset (1216/2748) and the zygote transcriptome contained 64% of our oogenic dataset (1117/1732), which represent 45% and 23% of the sperm and zygote transcriptomes (Figure 4, D and E). These comparisons therefore provide additional validation for our spermatogenic, oogenic, and gender-neutral datasets.

#### Datasets for differential exon usage in spermatogenic vs. oogenic gonads:

To further characterize spermatogenic and oogenic transcriptomes, we analyzed the original Seq data to look for transcript level differences. Out of 99,984 total exon–exon junctions identified by TopHat in both transcriptomes, 86,452 (86.5%) represented canonical junctions (consecutive exons) and 13,532 (13.5%) represented noncanonical junctions (nonconsecutive exons) (Table S3). Transcripts were assembled in each gonad transcriptome using Cufflinks. We detected 25,461 expressed transcripts in spermatogenic gonads and 24,333 expressed transcripts in oogenic gonads, including isoforms in both cases (Table S4).

**Figure 5:**
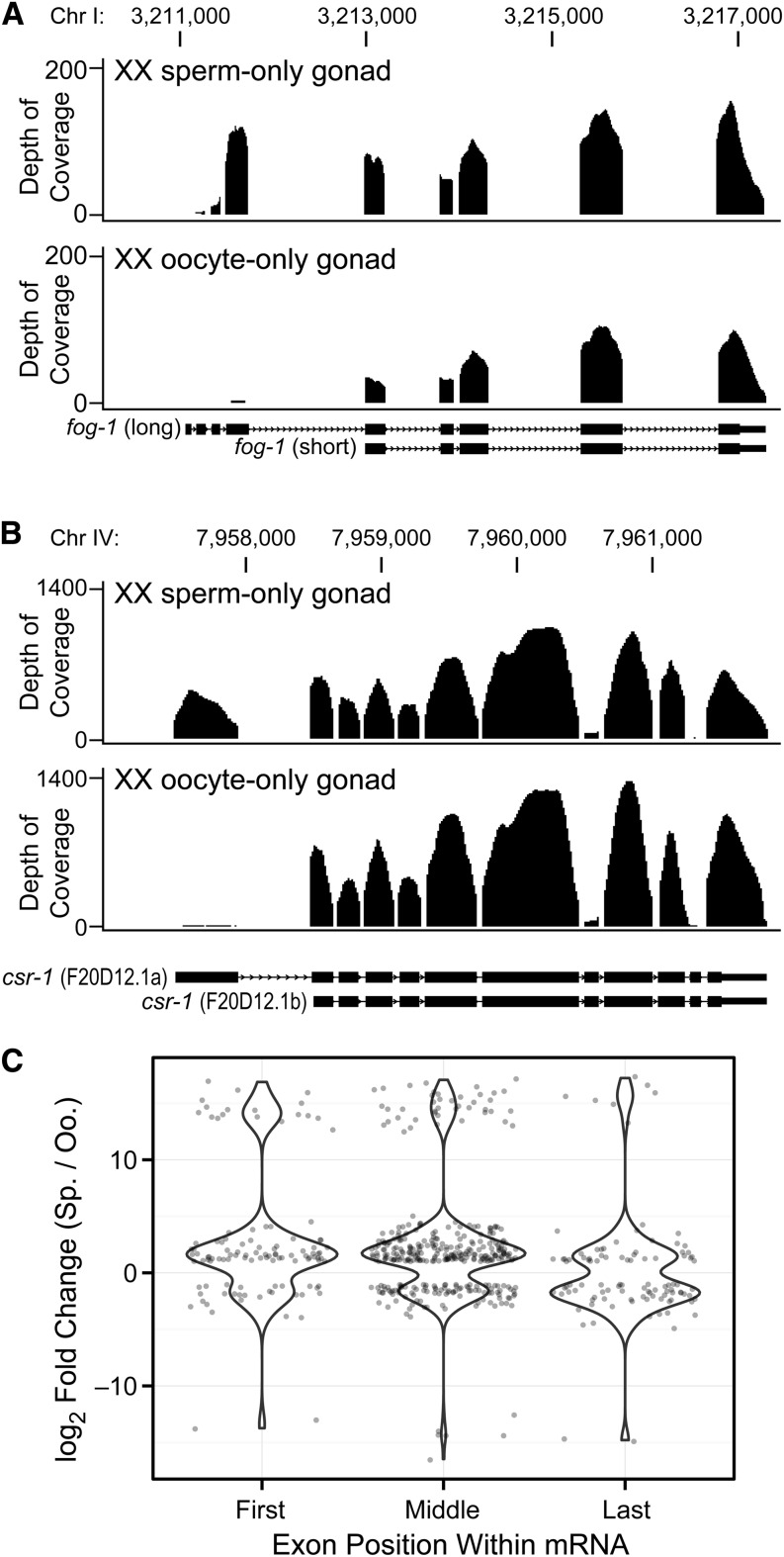
Sex-enriched exon usage in sperm-only *vs.* oocyte-only XX gonads. (A and B) Coverage plots of sequencing reads mapping to exons in spermatogenic gonads (above) and oogenic gonads (below). Depth of coverage is measured as average reads per nucleotide. Relevant isoform models are shown for reference at bottom. Smoothing windows: 5 pixels in (A) and 10 pixels in (B). (A) Differential exon usage at the *fog-1* locus. (B) Differential exon usage at the *csr-1* locus. (C) Locations within transcripts of differentially used exons. Exons are displayed according to log_2_ fold change of normalized counts in *fem-3(q96gf) vs. fog-2(q71)*. Positive values show increased exon use in sperm-only gonads, whereas negative values show increased exon use in oocyte-only gonads.

To identify isoforms enriched in spermatogenic and oogenic gonads, we analyzed differential exon usage between the gonadal transcriptomes. We identified 577 differentially expressed exons (DEXSeq; FDR < 1%) affecting 351 genes (Table S5). Of the 351 genes, 326 genes (93%) have annotated gene models on WormBase that correspond to our exon usage analysis. We also found that 253 genes (73%) were affected in protein-coding exons. As an example, we visualized mapped reads to the *fog-1* gene locus with the UCSC Genome Browser. In agreement with the exon usage analysis, we found both the long and short *fog-1* isoforms with enrichment of the “long” isoform in spermatogenic gonads and enrichment of the “short” isoform in oogenic gonads (Figure 5A). This finding mirrors *fog-1* isoforms found previously using Northern blots (Luitjens *et al.* 2000). We also identified, as another example, sex-specific enrichment for the first exon of the mRNA encoding Argonaute protein CSR-1 (Figure 5B), which is intriguing given the role of CSR-1 in spermatogenesis (Conine *et al.* 2013). Among the 351 genes with sex-enriched exons, 50% occurred in spermatogenic, 30% occurred in oogenic, and 17% occurred in gender-neutral genes (Table S5). To further characterize these 351 mRNAs, we performed Gene Ontology (GO) analysis. The following biological processes were over-represented compared with all gonad-expressed genes: cell cycle (*P* < 0.005); glucose metabolism (*P* < 0.007); and post-transcriptional gene regulation (*P* < 0.04). Molecular functions relating to ATP binding were also enriched (*P* < 0.003).

We next located differentially expressed exons within their transcripts: 19.2% (111/577) were the first exon; 17.7% (102/577) were the last exon; and 63.1% (364/577) were in the middle (Figure 5C). These middle exons reveal exon-skipping events predicted to alter the proteomes of spermatogenic *vs.* oogenic gonads. Although splicing factors have dramatic effects on gamete specification (Puoti and Kimble 1999, 2000; Kerins *et al.* 2010), the affected RNAs are not yet known. This dataset of differently expressed exons in spermatogenic and oogenic gonads may be a useful resource for finding events relevant to germline development or more broadly for studies of alternative 5′ and 3′ end formation as well as alternative splicing.

## Conclusions

This work provides new datasets for spermatogenic, oogenic, and gender-neutral genes expressed in the *C. elegans* gonad. The major advantages over earlier datasets are doubling the number of genes found expressed in the gonad, with most likely expressed in germline tissue, and identification of sex-enriched mRNA isoforms.

## Supplementary Material

Supporting Information
