# Supplementary material for: A New Dataset of Spermatogenic vs. Oogenic Transcriptomes in the Nematode Caenorhabditis elegans
Source: G3 (Bethesda). 2014 Jul 24;4(9):1765–72. doi: 10.1534/g3.114.012351 (PMC4169169; doi:10.1534/g3.114.012351)
Supplement: Supporting Information [file supp_4_9_1765__index.html]

A New Dataset of Spermatogenic vs. Oogenic Transcriptomes in the Nematode Caenorhabditis elegans — Supporting Information 

# A New Dataset of Spermatogenic *vs.* Oogenic Transcriptomes in the Nematode *Caenorhabditis elegans*

## Supporting Information for Ortiz *et al.*, 2014

**Files in this Data Supplement:**

- Table S1 - Genes expressed in the gonad determined by DESeq. NA = not applicable, either because gene was not in list being analyzed or because *in situ* data was not available for gene in question. (.xlsx, 1 MB)
- Table S2 - Genes expressed in the gonad with meiotic related functions. (.xls, 85 KB)
- Table S3 - Analysis of exon junctions in spermatogenic and oogenic gonads. (.xlsx, 4 MB)
- Table S4 - Transcripts expressed in gonads determined by Cufflinks. *q71* refers to *fog-2(q71)* oogenic gonads; *q96* refers to *fem-3(q96gf)* spermatogenic gonads. (.xls, 4 MB)
- Table S5 - Exon usage in mRNAs expressed in the gonad determined by DEXSeq. *q71* refers to *fog-2(q71)* oogenic gonads; *q96* refers to *fem-3(q96gf)* spermatogenic gonads. (.xls, 186 KB)
